# RNF122: A novel ubiquitin ligase associated with calcium-modulating cyclophilin ligand

**DOI:** 10.1186/1471-2121-11-41

**Published:** 2010-06-17

**Authors:** Zhi Peng, Taiping Shi, Dalong Ma

**Affiliations:** 1 Chinese National Human Genome Center, #3-707 North YongChang Road BDA, Beijing 100176, PR China; 2 Laboratory of Medical Immunology, School of Basic Medical Science, Peking University Health Science Center, 38# Xueyuan Road, Beijing, 100191, PR China; 3 Peking University Center for Human Disease Genomics, 38# Xueyuan Road, Beijing, 100191, PR China; 4 Department of Medical Oncology, Yijishan Hospital, Wannan Medical College, Wuhu, Anhui, 241001, PR China

## Abstract

**Background:**

RNF122 is a recently discovered RING finger protein that is associated with HEK293T cell viability and is overexpressed in anaplastic thyroid cancer cells. RNF122 owns a RING finger domain in C terminus and transmembrane domain in N terminus. However, the biological mechanism underlying RNF122 action remains unknown.

**Results:**

In this study, we characterized RNF122 both biochemically and intracellularly in order to gain an understanding of its biological role. RNF122 was identified as a new ubiquitin ligase that can ubiquitinate itself and undergoes degradation in a RING finger-dependent manner. From a yeast two-hybrid screen, we identified calcium-modulating cyclophilin ligand (CAML) as an RNF122-interacting protein. To examine the interaction between CAML and RNF122, we performed co-immunoprecipitation and colocalization experiments using intact cells. What is more, we found that CAML is not a substrate of ubiquitin ligase RNF122, but that, instead, it stabilizes RNF122.

**Conclusions:**

RNF122 can be characterized as a C3H2C3-type RING finger-containing E3 ubiquitin ligase localized to the ER. RNF122 promotes its own degradation in a RING finger-and proteasome-dependent manner. RNF122 interacts with CAML, and its E3 ubiquitin ligase activity was noted to be dependent on the RING finger domain.

## Background

The ubiquitin-proteasome system is involved in protein degradation and many biological processes such as transcription, cell cycle progression, antigen processing, cellular defense, signaling, and apoptosis [1,2]. In the ubiquitin-proteasome pathway, a ubiquitin-activating enzyme (E1) activates ubiquitin (Ub) by attaching it to a substrate via a thiol-ester linkage and then transferring the complex formed to the active-site cysteine of a ubiquitin carrier protein (E2). Formation of isopeptide bonds between the C terminus of Ub and the lysines on the substrate is catalyzed by a ubiquitin ligase (E3), which binds the substrate and catalyzes the transfer of Ub from a specific E2 to the substrate. The formation of a chain of Ub molecules on the substrate generally targets it for degradation by the 26 S proteasome [3]. Comparative genome analysis has revealed the presence of a few genes encoding E1, others encoding E2, and hundreds encoding E3 ligases [4].

RING finger proteins contain a RING finger domain, which was first identified as being encoded by the Really Interesting New Gene in the early 1990 s [5]. The RING finger domain contains 8 metal-binding residues that coordinate 2 zinc atoms in an interleaved pattern to facilitate correct folding, which is necessary for the biological actions of these proteins. Many RING finger proteins have been identified as ubiquitin ligases and are known to play an important role in various physiological processes. For example, MDM2, a representative RING finger protein, is a ubiquitin ligase of p53 [6]. Further, Cbl is known to play an important role in the ligand-induced ubiquitination of epidermal growth factor receptor (EGFR) via a mechanism that involves interaction between the RING finger domain and UbcH7 [7]. The tumor necrosis factor (TNF) receptor associated factors (TRAFs) contain a RING finger domain at the N-terminal region; TNFs play an important role in both adaptive and innate immunity [8]. However, there are still hundreds of RING finger proteins whose functions have yet to be characterized.

A cell-based screening performed in our laboratory revealed that the *RNF122 *gene is associated with cell viability [9]. *RNF122 *is expressed in several normal tissues and in tumor tissues and cell lines; RNF122 has been localized to the endoplasmic reticulum (ER) and the Golgi apparatus [10]. Comparative genomic hybridization (CGH) has revealed that *RNF122 *is overexpressed in anaplastic thyroid cancer cells [11]. However, RNF122 expression is not invariably detected in mammalian and *Escherichia coli *cells. The presence of the RING finger domain in RNF122 implies that RNF122 may be involved in the ubiquitylation pathway; however, to date, there has been no study that has sought to biochemically characterize of RNF122. Moreover, the mechanism of apoptosis induction mediated by RNF122 remains unclear. Given the importance of the biological functions of the RING finger proteins and the overexpression of RNF122 in anaplastic thyroid cancer cells, a functional characterization of this gene is highly warranted. The present study provides evidence that RNF122 is a new uncharacterized ubiquitin ligase. Further, we demonstrate that RNF122 interacts with CAML.

## Results

### Characterization of RNF122 expression in mammalian cells and *E. coli*

**Figure 1:**
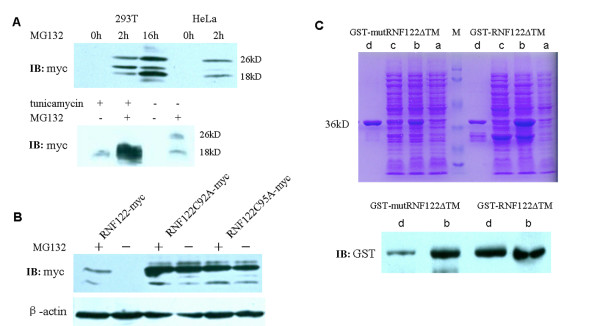
**Characterization of RNF122 expression in mammalian and *E. coli *cells. **(A). HEK293T and HeLa cells were transiently transfected with RNF122-myc. At 24 h post-transfection, the cells were treated with 2.5 μg/mL MG132 or 50 μg/mL tunicamycin and harvested at the indicated time. The cell lysates were subjected to SDS-PAGE and analyzed by western blotting using anti-myc and anti-β-actin antibodies. (B). HEK293T cells were transiently transfected with RNF122-myc or with RNF122C92A-myc or RNF122C95A-myc mutants. At 24 h post-transfection, the cells were treated with 50 μg/mL MG132, harvested after 6 h, and then analyzed by western blotting. (C). GST-RNF122ΔTM or GST-RNF122-ΔTM mutant proteins were expressed in *E. coli *and purified by affinity chromatography. The proteins were verified by SDS-PAGE and western blotting using an anti-GST primary antibody. a: pre-induction; b: post-induction; c: effluent obtained after affinity chromatography; d: purified protein.

Yu found that epitope-tagged RNF122 was not successfully expressed in mammalian and bacterial cells [11]. In the present study, we performed similar experiments and obtained the same results. However, northern blotting, RT-PCR, and subcellular localization studies have demonstrated the existence of *RNF122 *[10]. Many RING finger proteins are ubiquitin ligases that ubiquitinate themselves to facilitate their degradation by the ubiquitin proteasome. We accordingly examined the expression of RNF122 in HeLa and HEK293T cells after treatment with one of the ubiquitin proteasome inhibitors, namely, MG132. As illustrated in Figure 1A, RNF122 was detected in the MG132-treated cells. This result demonstrates that RNF122 is a substrate of the ubiquitin proteasome system. To verify the importance of the RING finger domain in the stability of RNF122, a mutant *RNF122 *gene was constructed using PCR-based mutagenesis and transfected into HEK293T cells. Subsequently, the total protein was extracted and analyzed by western blotting. The mutant RNF122 was detected in cells not treated with MG132; further, its expression was not significantly affected by MG132 treatment (Figure 1B). These results suggest that the RING finger domain is critical for the degradation of RNF122. Figure 1A and 1B shows 2 bands of approximately 18 kD and 26 kD, indicating that RNF122 may be glycosylated; this result is consistent with the fact that the RNF122 sequence contains some potential N-linked glycosylation sites (NxT/S). Moreover, treatment of the cells with tunicamycin, which inhibits N-linked glycosylation, caused a shift in the protein bands (Figure 1A). These results indicate that RNF122 is an N-linked glycosylated protein containing a RING finger that catalyses its own degradation in a proteasome-dependent manner.

We constructed a full-length GST-RNF122 fusion protein, but we were unable to demonstrate successful expression of this protein in bacteria (data not shown), which is consistent with the results obtained by Yu [11]. The transmembrane sites, usually represented by a hydrophobic sequence, are not expressed in *E. coli*. Hence, we constructed two truncated mutants of GST-RNF122, namely, GST-RNF122ΔTM and GST-mutRNF122ΔTM, in which the transmembrane (TM) domain was deleted. As illustrated in Figure 1C, the expression of both GST-RNF122ΔTM and GST-mutRNF122ΔTM was inducible. These proteins were purified successfully by affinity chromatography and confirmed by western blotting using an anti-GST antibody.

### In vivo and in vitro ubiquitination of RNF122

**Figure 2:**
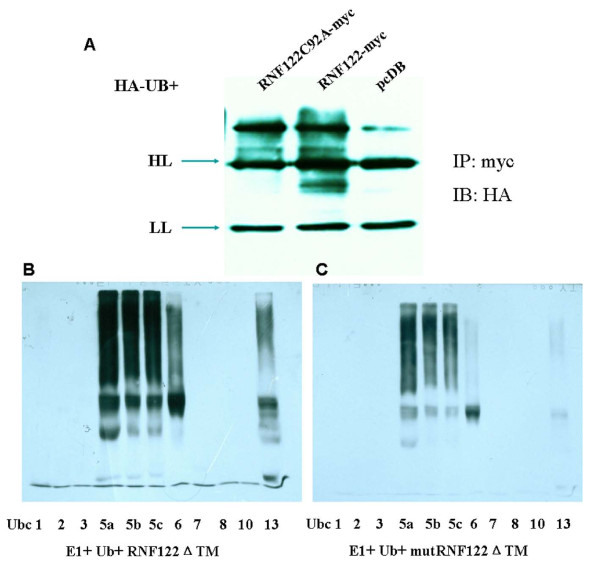
**In vivo and in vitro ubiquitylation of RNF122**. (A) HEK293T cells were seeded into 60-mm plates and transfected with 5 μg of Ub-HA and 5 μg of pcDB (empty) or wild-type or mutant RNF122-myc expression plasmid. At 48 h post-transfection, cell lysates in RIPA buffer were immunoprecipitated with anti-myc antibody agarose beads. The immunoprecipitates were analyzed on an immunoblot for HA. A smeared band was observed in the RNF122-myc group. (B) E2-selective ubiquitin ligase activity of RNF122. Bacterially expressed GST-RNF122ΔTM was incubated with ubiquitin, E1, and various E2 enzymes. Autoubiquitylation of RNF122 was detected by HRP-streptavidin immunoblotting. (C) RNF122 E3 ligase activity required an intact RING finger domain. The reactions were performed using ubiquitin, E1, E2 (UbcH5b or UbcH5c), and E3 (GST-RNF122ΔTM, or mutant GST-RNF122ΔTM), as indicated.

To further examine the ubiquitination of RNF122, we performed in vivo ubiquitination studies. We coexpressed RNF122-myc with HA-ubiquitin in HEK293T cells, recovered the immunoprecipitates using anti-myc antibody, and performed immunoblotting using the anti-HA antibody. As shown in Figure 2A, a smeared band, which indicates ubiquitination, appeared in the blots of lysates of cells cotransfected with *RNF122-myc *and *HA-ubiquitin*, but this was not detected in the vector control group. Hence, we confirmed that RNF122 undergoes ubiquitination in vivo. Further, we performed the same experiment using an RNF122 mutated in the RING finger domain and found that this mutant (RNF122C92A-myc) was also ubiquitylated in vivo, as evident from the high-molecular-weight smeared band detected on blots of the lysates of the cells coexpressing RNF122C92A-myc and ubiquitin (Figure 2A). However, the degree of ubiquitination of this mutant protein was significantly lower than that of RNF122-myc; a finding that is consistent with the proteasome degradation of the wild-type and mutant RNF122.

The presence of the RING finger domain in RNF122 confers the properties of a ubiquitin ligase to this protein. To investigate whether RNF122 has ubiquitin ligase activity, we established an in vitro autoubiquitination assay, which was performed according to the manufacturer's instructions. Briefly, E1, E2, and bacterially expressed GST-RNF122ΔTM were added to a reaction mixture containing ubiquitin and ATP. We treated the RNF122 fusion proteins with different E2 enzymes to determine which E2 supports RNF122-mediated ubiquitination. As is evident from Figure 2B, polyubiquitination was detected only in cells treated with the following E2-conjugating enzymes: UbcH5a, UbcH5b, UbcH5c, Ubc6, and Ubc13. To clarify the role of the RING finger domain in ubiquitination, we performed an auto ubiquitination assay using RNF122 containing a single-site mutations (Cys92Ala) in the RING finger domain (GST-RNF122C92A-ΔTM). As shown in Figure 2C, the mutation compromised the autoubiquitylation activity of UbcH5a, UbcH5b, UbcH5c, Ubc6, and Ubc13. These results demonstrate that RNF122 is an E3 ligase that is selective for UbcH5a, UbcH5b, UbcH5c, Ubc6, and Ubc13.

### RNF122 interacts with CAML

**Figure 3:**
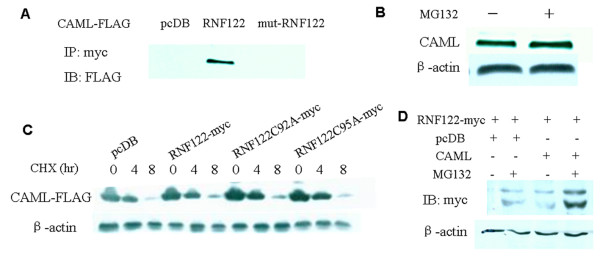
**Interaction of RNF122 with CAML**. (A) In mammalian cells, RNF122 interacted with CAML. HEK293T cells were seeded into 60-mm plates and transfected with pcDB (empty), RNF122-myc or mut-RNF122-myc vectors in conjunction with CAML-FLAG. After 48 h, the cells were treated with 2.5 μg/mL MG132 for 6 h, lysed in PBS containing 1% Triton X-100, and immunoprecipitated with anti-myc antibody agarose beads. (B) CAML is not involved in the proteasome-dependent degradation pathway. HEK293T cells were treated with 2.5 μM MG132 or vehicle (DMSO) for 6 h. The cells were then harvested and the expression of CAML was analyzed by western blotting using an anti-CAML antibody. The membrane was subsequently reprobed with an anti-β-actin antibody. (C) HEK293T cells were transiently cotransfected with CAML-FLAG and pcDB, RNF122-myc, RNF122C92A-myc, or RNF122C95A-myc. At 24 h post-transfection, the cells were treated with 50 μg/mL CHX and harvested at the indicated times. The cell lysates were subjected to SDS-PAGE and analyzed by western blotting by using antisera against FLAG and β-actin. (D) The level of RNF122 was found to increase in a proteasome-independent manner when it was coexpressed with CAML. HEK293T cells were transfected with empty (pcDB) or CAML expression vector along with RNF122-myc, and subsequently treated with 2.5 μg/mL MG132 or DMSO at 24 h post-transfection. After 24 h, the cell lysates were suspended in PBS containing 1% Triton X-100 and a protease inhibitor cocktail, and then immunoblotted with an anti-myc antibody.

Next, the yeast two-hybrid system was used to identify the potential substrates of RNF122. The experiments were performed by Ruixing Corp. (Shanghai, China). The results revealed that RNF122 interacts with CAML, a protein associated with calcium signaling and T cell activation. To confirm the interaction between RNF122 and CAML, we co-expressed these proteins in HEK293T cells and immunoprecipitated RNF122-myc with the anti-myc antibody. Western blot analysis of the immunoprecipitate obtained using the anti-FLAG antibody revealed that RNF122 but not its mutant form was coprecipitated with FLAG-tagged CAML (CAML-FLAG), demonstrating their association in mammalian cells (Figure 3A). The results also suggested that CAML interacts with RNF122 through the RING finger domain. Hence, the RING finger protein functions as a ubiquitin ligase that ubiquitinylates the substrate and targets it to the ubiquitin proteasome system. We accordingly performed experiments to determine whether CAML is a substrate of RNF122 and whether it can be degraded by the ubiquitin proteasome system. As shown in Figure 3B, however, CAML levels in MG132-treated cells were not elevated, suggesting that CAML may not be involved in the proteasome-dependent degradation pathway. Furthermore, CAML protein levels remained unchanged even when CAML-FLAG and RNF122-myc or mutant RNF122 were coexpressed (Figure 3C). Overall, we conclude that CAML interacts with RNF122 but is not a substrate for its ubiquitylating activity. Interestingly, RNF122 expression was occasionally detected in cells that overexpressed CAML. As illustrated in Figure 3D, RNF122 was stabilized when CAML was overexpressed. Hence, it can be speculated that the interaction between CAML and RNF122 inhibits the ubiquitination of RNF122, and thereby stabilizes RNF122.

### Colocalization of RNF122 and CAML in HEK293T and HeLa cells

**Figure 4:**
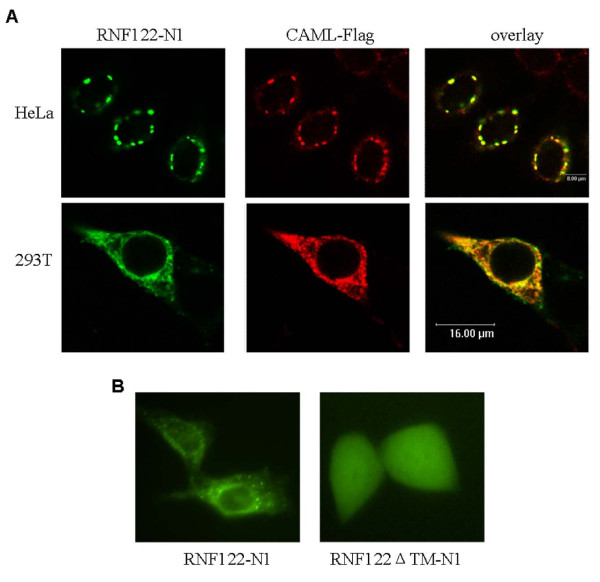
**Subcellular localization of RNF122**. (A) HeLa or HEK293T cells were grown on coverslips and transfected with RNF122-N1 and CAML-FLAG. To inhibit the degradation of RNF122-N1, the cells were treated with 2.5 μM MG132 for 6 h. The cells were then treated with anti-FLAG antibody and FITC-conjugated goat anti-mouse antibody, and observed under a confocal microscopy. The results showed that the cytoplasmic distribution of CAML entirely overlapped that of RNF122. (B) HEK293T cells were transfected with RNF122-N1 and RNF122ΔTM-N1 and observed under a fluorescence microscope.

In order to confirm the intracellular interaction between RNF122 and CAML, we constructed a plasmid, namely, pEGFP-N1-RNF122, encoding green fluorescent protein, and a CAML-FLAG-encoding plasmid and cotransfected these into HeLa cells or HEK293T cells. As shown in Figure 4A, both pEGFP-N1-RNF122 and CAML-FLAG were observed to be localized in the cytoplasm and were highly concentrated in spots around the nucleus; moreover, the distribution of CAML was similar to that of RNF122. Further experiments on the localization of CAML yielded the same result, namely, that CAML is partially localized to the ER. This observation is consistent with our previous finding that RNF122 is localized to the ER. These data confirmed that RNF122 and CAML interact to form a complex that is functional in the cytoplasm.

### The TM domain is critical to the localization of RNF122

RNF122 contains a putative TM domain near the N terminus (amino acid residues 37-59). To investigate whether this domain is required for the localization of RNF122 to the ER, we generated a truncated mutant of RNF122 in which 60 N-terminal amino acids were deleted. The expression vectors pcDB/RNF122-ΔTM and RNF122-ΔTM-GFP were constructed and used to evaluate the functions and subcellular localization of the truncated RNF122. The cytoplasmic expression pattern of the RNF122 mutant was noted to be diffuse as compared to the expression pattern of the wild-type RNF122 (Figure 4B), indicating that the N-terminal TM domain of RNF122 is required for its specific localization. These findings suggest that the putative N-terminal TM domain is necessary for the localization of RNF122.

## Discussion

The importance of the RING finger domain in ubiquitination is supported by the fact that several RING finger proteins are associated with Ubcs and/or specific proteins destined for proteasome-dependent degradation, although there have been no reports confirming their E3 activities toward these proteins.

In our previous study, we performed northern blotting and RT-PCR analysis, and confirmed that RNF122 is widely expressed in several tissues and cell lines and that it is localized to the ER and the Golgi apparatus. A cell-based screening test revealed that RNF122 can downregulate the expression of the prolactin (*PRL*) gene. Further studies showed that RNF122 was associated with the viability of HEK293T cells. However, the exact biological function of RNF122 is still unknown. In the present study, we characterized the biological role of RNF122. RNF122 contains a RING domain, which suggests that it catalyzes its own degradation. Furthermore, through in vivo and in vitro ubiquitination assays, RNF122 was identified as a new ubiquitin ligase. We also found that the TM domain of RNF122 is critical for its localization to the ER.

CAML is a ubiquitous protein containing 296 amino acids. It contains 3 putative TM domains near its C terminus and has been shown to be a resident protein of the ER [12,13]. There are several proteins that can interact with CAML, including CAML interactor (TACI) [14], epidermal growth factor receptor (EGFR) [15], p56^lck ^[16], Kaposi's sarcoma virus-associated mitochondrial K7 protein [17], type-1 angiotensin II receptor-associated protein (ATRAP) [18], and γ2 subunit-containing gamma-aminobutyric acid type A receptor (GABA_A_R) [19]. Consistent with the findings of the previous studies on CAML, our findings showed that RNF122 interacts with CAML. The coimmunoprecipitation and colocalization experiments verified the interaction between RNF122 and CAML in mammalian cells and, at the same time, showed that overexpression of RNF122 does not reduce the CAML levels. Nonetheless, it seems unlikely that CAML is a substrate for the ubiquitin ligase activity of RNF122. This supposition is based on the observation that CAML levels were not reduced by the coexpression of RNF122 and were not increased in MG132-treated cells.

We have validated the interaction between CAML and RNF122 in vivo when the proteins are overexpressed, which may not necessarily represent the endogenous condition. However, we have failed to testify endogenous interaction because of the limited RNF122 expression and its poor antibody. In the stability study, RNF122 can be stabilized when CAML was overexpressed, which give a clue that RNF122 and CAML are functional related. It has been concluded that CAML has been proposed to regulate trafficking of EGFR and the GABA_A _receptor. The stalibility of EGFR and GABA_A _receptor can be modulated through c-Cbl and Plic-1 respectively, and they both are involved in ubiquitin-proteasome system [20,21]. So we speculate that RNF122 may regulate EGFR and GABA_A _receptor stability through ubiquitin-proteasome system. We are still focus on the biological role of RNF122 in the next investigation. The results may give us a new direction to clarify the biological function of RNF122.

Previous study show that CAML can interact with EGFR in ligand-dependent way, which gave us a clue that RNF122 may involved in EGFR pathway. We have also found that RNF122 effect on cell viability may relate to ERK pathway (data not shown). However, the precise molecular mechanisms such as the interaction between EGFR and RNF122 still need to be further explored.

## Conclusions

In conclusion, RNF122 can be characterized as a C3H2C3-type RING finger-containing E3 ubiquitin ligase localized to the ER. RNF122 promotes its own degradation in RING finger-and proteasome-dependent manner. RNF122 interacts with CAML, and its E3 ubiquitin ligase activity was noted to be dependent on the RING finger domain. Further studies are required to clarify the molecular mechanism, such as substrate binding, that underlie RNF122 action.

## Methods

### Constructs and transfection

Full-length cDNA of *RNF122 *(GenBank accession no. NM_024787.2) was obtained and pEGFP-N1-RNF122 was constructed according to previously described methods [10](Wang, Shi et al. 2006). *RNF122-myc *was amplified and then ligated into pcDNA.3.1/myc-His(-)B (Invitrogen, USA). The first or the second cysteine residue of the RING finger was substituted with an alanine residue by polymerase chain reaction (PCR)-based site-directed mutagenesis, which resulted in the formation of products designated as RNF122C92A-myc or RNF122C95A-myc, respectively. We get RNF122-ΔTM-GFP and CAML-FLAG by inserting RNF122-ΔTM and CAML into the pEGFP-N1 and pFLAG-CMV2 vectors (Clontech, USA), respectively, using a PCR-based method. The bacterially expressed GST-RNF122ΔTM or GST-mutRNF122ΔTM fusion proteins (GST; glutathione S-transferase) were obtained using PCR fragments encoding residues 61-155 of human RNF122 or RNF122-C92A-myc. The PCR primers contained *Xho*I and *Bam*HI sites. The resulting PCR fragment was inserted into the pGEX-4T-1 vector (GE Healthcare, USA). All the insertions were confirmed by DNA sequencing.

DNA transfection was performed using VigoFect (Vigorous, China), a non-liposomal cationic formula, according to the manufacturer's instructions.

### Prokaryotic expression

*E. coli *strain BL-21(DE3) (Novagen, Madison, WI) were cultured overnight at 37°C and induced with 0.4 mM isopropyl-1-thio-β-D-galactopyranoside for 1 h at room temperature (RT). The bacterial pellets were resuspended in a solution containing 50 mM Tris (pH 7.4), 1 mM ethylenediaminetetraacetic acid (EDTA), 1% Triton X-100, 5 mM dithiothreitol (DTT), and 2 mM phenylmethylsulfonyl fluoride (PMSF) (sonication buffer), and lysed by probe sonication using 4 mL of sonication buffer per 100 mL of bacterial culture. The sonicate was clarified by centrifugation at 4°C for 15 min at 18,000 × *g*, divided into aliquots, and stored at -70°C. To estimate the level of GST fusion proteins expressed, the sonicates were incubated with glutathione-Sepharose (GS) beads, washed, and subjected to sodium dodecyl sulfate-polyacrylamide gel electrophoresis (SDS-PAGE); subsequently, the gel was stained with Coomassie Brilliant Blue R-250.

### Cell lines and reagents

Human embryonic kidney cell line HEK293T and human cervix carcinoma cell line HeLa were obtained from the American Type Culture Collection (Manassas, VA). HEK293T and HeLa cells were cultured (37°C, 5% CO_2 _humidified atmosphere) in Dulbecco's modified Eagle's medium (DMEM) (Invitrogen, Carlsbad, CA) containing 10% fetal bovine serum (FBS) (Hyclone, Logan, UT) and supplemented with 2 mM L-glutamine (Invitrogen, Carlsbad, CA). Monoclonal mouse antibodies against β-actin, c-myc, and FLAG were purchased from Sigma (Sigma-Aldrich, St Louis, MO); horseradish peroxidase (HRP)-streptavidin and HRP-myc antibodies, from Upstate (USA); MG132, from Sigma (Sigma-Aldrich, St Louis, MO); tunicamycin, from Alexis (USA); cycloheximide, from Calbiochem (USA); and a ubiquitin-conjugating enzyme kit (mammalian) from Biomol (USA).

### Immunoprecipitation and immunoblotting

HEK293T cells were transiently transfected with the epitope-tagged constructs using VigoFect, as described above. Forty-eight hours after transfection, the cells were washed 3 times in phosphate-buffered saline (PBS), harvested by scraping, and centrifuged (5 min, 500 × *g*). The pelleted cells were homogenized in a cell lysis buffer [50 mmol/L Tris-HCl (pH 7.4), 150 mmol/L NaCl, 5 mmol/L EDTA-Na_2_, 1% NP-40; 1 μg/μL pepstain, 1 μg/μL aprotinin, 1 μg/μL leupeptin, 1 mmol/L DTT, and 0.0174 μg/μL PMSF]. The cell lysates thus obtained were centrifuged (20 min, 14,000 × *g*, 4°C), and the resulting supernatants were combined with 12.5 μL (packed gel) of either anti-c-Myc or anti-FLAG M2 affinity agarose (Sigma-Aldrich, St Louis, MO), mixed, and incubated in 4°C overnight. The immunoadsorbents were recovered by centrifugation (5 min, 700 × *g*) and washed 3 times with a high-salt buffer [500 mmol/L Tris-HCl (pH 7.4), 150 mmol/L NaCl, 5 mmol/L EDTA-Na_2_, 1% NP-40, 1 μg/μL pepstain, 1 μg/μL aprotinin, 1 μg/μL leupeptin, 1 mmol/L DTT, and 0.0174 μg/μL PMSF], followed by re-suspension in cell lysis centrifugation (5 min, 700 × *g*), and the same procedure was repeated using PBS. The samples were then eluted using 60 μL of 2 × SDS loading buffer (Sigma-Aldrich, St Louis, MO) and analyzed by western blotting using anti-myc or anti-FLAG antibodies. Subsequently, the bands were detected using Ig-HRP-conjugated antibodies and an enhanced chemiluminescence (ECL) detection system (GE Healthcare, USA).

### Confocal microscopy

The HEK293T or HeLa cells were cotransfected with pEGFP-N1-RNF122 and CAML22-N1 CCAML-FLAG, grown on coverslips, fixed using 3.7% paraformaldehyde at RT for 30 min, and then permeabilized with 0.1% Triton X-100 at RT for a further 10 min. The cover slips were washed 3 times with PBS, treated with a blocking buffer (5% bovine serum albumin in PBS) for 30 min, and incubated with an anti-FLAG primary antibody for 2 h at RT. The cells were then washed 3 times (10 min each) in PBS and incubated with a secondary antibody for 1 h at 37°C. Rhodamine-conjugated goat anti-mouse immunoglobulin G (IgG; ZhongShan Biotechnology) was used as the secondary antibody.

### In vivo ubiquitination

In order to analyze ubiquitination, we transfected the cells with a myc-tagged RNF122 vector and hemagglutinin (HA)-tagged ubiquitin, as described previously. Next, we harvested the cells and incubated them with 1 volume of 2% SDS in TBS (10 mM Tris-HCl, pH 8.0) at 95°C for 10 min in order to achieve lysis. Subsequently, we added 9 volumes of 1% Triton X-100 and 2 mM EDTA in TBS to the cell lysates, and incubated the lysates on ice for 1 h. The protein concentrations of the lysates were determined by the bicinchoninic acid (BCA) assay. For immunoprecipitation, 1 mg of protein was incubated with the anti-myc antibody at 4°C overnight; subsequently, this mixture was incubated with protein G beads for 2 h. The beads were washed twice with NaCl (1 M) in TBS supplemented with NP-40 (1%), β-mercaptoethanol (0.05%), and EDTA (1 mM). The proteins were loaded onto a 12% SDS-PAGE gel and analyzed by immunoblotting using the aforementioned antibodies and an ECL detection kit (GE Healthcare, USA).

### In vitro ubiquitination

In vitro ubiquitination was performed according to the instructions provided with the ubiquitination kit (BioMol, USA). Briefly, the assays were carried out at 30°C in a 50-μL reaction mixture containing 25 mM 4-(2-hydroxyethyl)-1-piperazineethanesulfonic acid (HEPES), 100 U/mL of isopentenylpyrophosphate (IPP) (Sigma-Aldrich, St Louis, MO), 50 mM DTT, 50 mM EDTA, 1 mM Mg-ATP (pH 7.5), 100 nM E1 enzyme, 1 μM E2 enzyme, 10 μM GST-RNF122-ΔTM (or mutant GST-RNF122-ΔTM) fusion protein, and 2.5 μM biotin-labeled ubiquitin. After 30-60 min, the reactions were terminated by the addition of a non-reducing gel loading buffer, and subsequently the protein components were separated by SDS-PAGE and analyzed by western blotting using the HRP-streptavidin antibody.

### Yeast Two-Hybrid Screening

Yeast Two-Hybrid Screening was performed at Shanghai Genomics (Shanghai, China). The two-hybrid screening system has been previously described [22]. Briefly, the library consisted of 1500 known genes associated with cell apoptosis, cell proliferation, and cell cycles. Each ORF was amplified by PCR using Pfu DNA polymerase and cloned into pGBK-RC, a Gal4 DNA-binding domain-based bait vector, and pGAD-RC, a Gal4 activation domain-based prey vector, following the MATCHMAKER GAL4 Two-Hybrid System 3 and Libraries User Manual PT3247-1 (PR94575) protocol (Clontech, Mountain View, CA). Plasmids with inserts of expected sizes were confirmed by colony PCR followed by agarose gel electrophoresis. RNF122 bait vector and prey vectors were cotransfected in yeast Y190 and spread into SD/-T-L-H. Formed colonies were picked out, cracked in liquefacient nitrogen, and subsequently utilized in colony lift filter assays.

## Authors' contributions

ZP participated in the design of the study, carried out the function research, performed the validation assays, and was pivotal in drafting the manuscript. DM and TS supervised the study and were involved in the conceptualization and writing. All authors read and approved the final manuscript.
